# Retraction: A rapid and novel method for the synthesis of 5-substituted 1*H*-tetrazole catalyzed by exceptional reusable monodisperse Pt NPs@AC under the microwave irradiation

**DOI:** 10.1039/d1ra90149d

**Published:** 2021-10-07

**Authors:** Muharrem Kaya, İbrahim Esirden

**Affiliations:** Biochemistry Department, Faculty of Arts and Science, Dumlupınar University, Evliya Çelebi Campus 43100 Kütahya Turkey; Chemistry Department, Faculty of Arts and Science, Dumlupınar University, Evliya Çelebi Campus 43100 Kütahya Turkey

## Abstract

Retraction for ‘A rapid and novel method for the synthesis of 5-substituted 1*H*-tetrazole catalyzed by exceptional reusable monodisperse Pt NPs@AC under the microwave irradiation’ by Esma Erken *et al.*, *RSC Adv.*, 2015, **5**, 68558–68564, DOI: 10.1039/C5RA11426H.

Muharrem Kaya and İbrahim Esirden hereby wholly retract this *RSC Advances* article due to concerns with the reliability of the data in the published article.

The high-resolution transmission electron micrograph inset in Fig. 2 that represents Md-Pt NPs@AC is the same as Fig. 2c in a *Chemistry Select* article by Haydar Göksu, Betül Çelik, Yunus Yıldız, Fatih Şen and Benan Kılbaş,^1^ which is a high-resolution transmission electron micrograph representing a CoPd NP. Fatih Sen provided replacement data for consideration. However, an expert reviewed the author’s response and concluded that it did not satisfactorily address the concerns, and that the replacement figure did not fully support the conclusions. Given the significance of the concerns about the validity of the data, the findings presented in this paper are no longer reliable.

Muharrem Kaya approves the retraction of this article due to concerns with the structural characterisation of the catalyst and therefore the conclusions presented may not be valid. Muharrem Kaya also states that the figures under question are out of their expertise and that they did not contribute to the data collection or processing of these figures. Muharrem Kaya contributed to the evaluation of the catalytic performances of the composite materials only.

Fatih Sen opposes this retraction. Esma Erken was contacted but did not respond.

Signed: Muharrem Kaya and İbrahim Esirden

Date: 23^rd^ September 2021

Retraction endorsed by Laura Fisher, Executive Editor, *RSC Advances*

## Supplementary Material

## References

[cit1] Göksu H., Çelik B., Yıldız Y., Şen F., Kılbaş B. (2016). Chemistry Select.

